# Semi-parametric empirical Bayes factor for genome-wide association studies

**DOI:** 10.1038/s41431-020-00800-x

**Published:** 2021-01-25

**Authors:** Junji Morisawa, Takahiro Otani, Jo Nishino, Ryo Emoto, Kunihiko Takahashi, Shigeyuki Matsui

**Affiliations:** 1 Department of Biostatistics, Nagoya University Graduate School of Medicine, Nagoya, Japan; 2 Department of Public Health, Graduate School of Medical Sciences, Nagoya City University, Nagoya, Japan; 3 Division of Bioinformatics, National Cancer Center Research Institute, Tokyo, Japan; 4 Medical and Dental Data Science Center, Tokyo Medical and Dental University, Tokyo, Japan; 5 Department of Data Science, The Institute of Statistical Mathematics, Tokyo, Japan

**Keywords:** Epidemiology, Genetics

## Abstract

Bayes factor analysis has the attractive property of accommodating the risks of both false negatives and false positives when identifying susceptibility gene variants in genome-wide association studies (GWASs). For a particular SNP, the critical aspect of this analysis is that it incorporates the probability of obtaining the observed value of a statistic on disease association under the alternative hypotheses of non-null association. An approximate Bayes factor (ABF) was proposed by Wakefield (Genetic Epidemiology 2009;33:79–86) based on a normal prior for the underlying effect-size distribution. However, misspecification of the prior can lead to failure in incorporating the probability under the alternative hypothesis. In this paper, we propose a semi-parametric, empirical Bayes factor (SP-EBF) based on a nonparametric effect-size distribution estimated from the data. Analysis of several GWAS datasets revealed the presence of substantial numbers of SNPs with small effect sizes, and the SP-EBF attributed much greater significance to such SNPs than the ABF. Overall, the SP-EBF incorporates an effect-size distribution that is estimated from the data, and it has the potential to improve the accuracy of Bayes factor analysis in GWASs.

## Introduction

Genome-wide association studies (GWASs) are comprehensive studies on the relationship between disease traits and single nucleotide polymorphisms (SNPs), throughout the genome, and have identified susceptibility gene variants for many complicated diseases [1, 2]. The data-analysis approach commonly used for identifying susceptibility gene variants in GWASs is statistical hypothesis testing based on the *P* value. Many authors, however, have pointed out that the *P* value has fundamental limitations [3]. A critical limitation is that the *P* value only conveys information about dissociation from the null hypothesis (null association), and it controls the probability of yielding a false positive based on the probability distribution of a test statistic under the null hypothesis, but not the probability of yielding a false negative. In GWASs, the lack of power due to the use of the extremely strict, genome-wide significance level [4, 5], 5 × 10^−8^, has also been criticized [6–8], as several studies have shown that many SNPs not reaching genome-wide significance are associated with various traits [9–11].

Thus far, increasing numbers of studies have used the Bayes factor (BF), in addition to the *P* value [12–14]. Typically, the BF is based on a sufficient statistic regarding the association between a disease and a particular SNP, and it compares the probability of observing a value of the statistic under the null hypothesis and the corresponding probability of observing this value under the alternative hypothesis. Thus, the BF conveys more information than the *P* value, since it takes into account not only the false positive, but also the false negative. This is particularly true in the Bayesian decision-theoretic testing [15]; the rejection of the null hypothesis based on the posterior odds ratio across the two hypotheses is transformed to a comparison of the BF and the threshold, expressed as the product of a prior odds ratio and the cost of the false negative relative to the false positive (see also “Hypothesis testing and the BF”).

When calculating the denominator of the BF, which represents the probability that the observed statistic value is under the alternative hypothesis, it is necessary to specify a prior distribution for an association parameter or effect size of a SNP (such as a coefficient of the log odds ratio in a logistic model), as well as nuisance parameters (such as an intercept coefficient in a logistic model), under the alternative hypothesis. Wakefield [16] sidestepped the specification of the prior distribution for the nuisance parameters and derived an explicit form of an approximate BF (ABF) for the association parameter of interest based on two approximations, (1) asymptotic normality of the estimated effect size and (2) a normal prior *N*(0, *W*) with the variance *W* for the effect-size distribution [16].

**Fig. 1:**
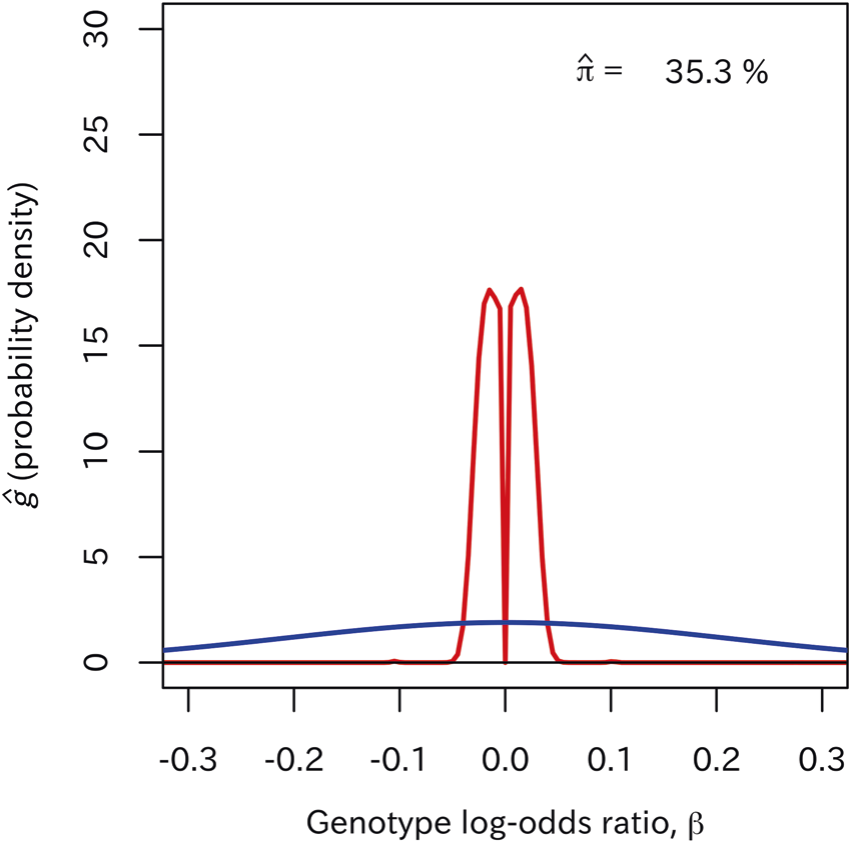
The estimated effect-size distribution used in the SP-EBF (red line) and the prior distribution *N*(0,*W*) with *W* = 0.21^2^ used in the ABF (blue line) in the bipolar disorder dataset.

However, the difficulty in specifying the prior distribution (as seen in many Bayesian analyses) also applies to the BF analysis. For the ABF, Wakefield proposed some specifications of the prior variance *W* [16]. For example, *W* can be specified as *W* = 0.21^2^ with a 95% belief that the effect size in terms of the odds ratio is within 1/1.5–1.5. More complex specifications incorporating dependence of the effect on the minor allele frequency (MAF) are also possible [16]. However, even with these arguments, there is always a risk of mis-specifying the prior distribution, especially in exploratory GWASs with limited prior information. To address misspecification of the variance *W*, some authors proposed to introduce a prior distribution for *W* [17] or to perform an empirical Bayes estimation of *W* [18]. However, normality of the effect-size distribution is a conventional assumption as there is no guarantee that it is reasonable. Some authors suggest the use of other parametric priors, such as Laplace priors [19]. Actually, the effect-size distribution is expected to have various distributional forms, reflecting complicated biological mechanisms between genetic factors and disease (see Figs. 1 and S4).

In this paper, we propose an empirical Bayes method with a flexible, nonparametric prior for the effect-size distribution to address the issue of misspecification. Our model is semi-parametric because of a combination of the nonparametric prior with the theoretically reasonable, asymptotic normality for the sampling distribution of the estimated effect size [8, 11, 20] (as done in the ABF). Even with the nonparametric prior, we can accurately estimate the effect-size distribution from high-dimensional genomic data, plausibly involving a large quantity of parallel data structures. See Nishino et al. [11] and Otani et al. [8] for the effectiveness of our estimation approach in the context of GWAS.

As such, our semi-parametric empirical BF (SP-EBF) method intends to improve the current BF analysis, possibly with inappropriate prior distributions. In other words, with the use of appropriate (nonparametric) prior distributions, our method aims to realize the inherent effectiveness of the BF analysis, potentially rendering it superior to traditional GWAS analysis based only on the *P* value.

## Methods

### Hypothesis testing and the BF

In a GWAS, each SNP is tested individually for its association with disease. Typically, the following univariate logistic regression model is assumed for the *j*th SNP,

$$\log \frac{{\eta _{ij}}}{{1 - \eta _{ij}}} = \alpha _j + \beta _jx_{ij},$$

where *η*_*ij*_ is the probability of disease for the *i*th subject with genotype *x*_*ij*_ (*x*_*ij*_ = 0, 1, or 2), and *α*_*j*_ and *β*_*j*_ are intercept and effect-size (log odds ratio) parameters [21] (*i* = 1, …, *n*; *j* = 1, …, *m*). When performing a test of the null hypothesis, *H*_0_:*β*_*j*_ = 0, a Wald *Z* value is expressed as follows, $z_j = \hat \beta _j/\sqrt {V_j} ,$ where $\hat \beta _j$ is a maximum likelihood estimate of *β*_*j*_, and *V*_*j*_ is an estimated variance of $\hat \beta _j$. Note that this test is a usual univariate test on single SNPs for common variants. Typical quality control processes remove low-frequency variants (e.g., MAF < 1%), so that the following BF analyses that are based on summary statistics $(\hat \beta _j,V_j)$ would not cover such rare variants.

The BF is defined by the ratio of the probability of observing $\hat \beta _j$ under *H*_0_ and the corresponding probability under an alternative hypothesis *H*_1_:*β*_*j*_ ≠ 0,

$${\mathrm{BF}}(\hat \beta _j) = \frac{{{\mathrm{Pr}}\left( {\hat \beta _j{\mathrm{|}}H_0} \right)}}{{{\mathrm{Pr}}\left( {\hat \beta _j{\mathrm{|}}H_1} \right)}}$$

For example, when the BF = 0.01, the obtained value $\hat \beta _j$ can be interpreted as being 100 times more likely to occur under the alternative hypothesis than under the null hypothesis.

For a particular SNP, a formal Bayesian decision-theoretic testing is to reject *H*_0_ if the posterior odds of *H*_0_, i.e., ${\mathrm{Pr}}\left( {H_0{\mathrm{|}}\hat \beta _j} \right)/\Pr \left( {H_1{\mathrm{|}}\hat \beta _j} \right)$, is smaller than the ratio of costs, $R = c_{{\mathrm{FN}}}/c_{{\mathrm{FP}}}$, where *c*_FP_ and *c*_FN_ are the costs of the false positive (type I error) and false negative (type II error), respectively [15]. As the posterior odds of *H*_0_ are expressed as a product of the BF and the prior odds of *H*_0_, that is, ${\mathrm{Pr}}\left( {H_0{\mathrm{|}}\hat \beta _j} \right)/\Pr \left( {H_1{\mathrm{|}}\hat \beta _j} \right) = {\mathrm{BF}} \times \left\{ {{\mathrm{Pr}}(H_0)/{\mathrm{Pr}}(H_1)} \right\}$, the aforementioned Bayesian decision-theoretic testing can be transformed to a decision rule to compare the BF with a relative cost *R* divided by the prior odds, i.e., if ${\mathrm{BF}}(\hat \beta _j) < R/(\Pr \left( {H_0} \right){\mathrm{/Pr}}(H_1)),$ then *H*_0_ is rejected.

### The ABF

The ABF was proposed by Wakefield [16]. In this analysis, for the *j*th SNP an approximation of asymptotic normality is employed for the estimate of *β*_*j*_, i.e., $\hat \beta _j$. Thus, under *H*_0_: *β*_*j*_ = 0, the distribution of $\hat \beta _j$ is specified as *N* (0, *V*_*j*_). Similarly, under the alternative hypothesis, *H*_1_:*β*_*j*_ ≠ 0, the distribution of $\hat \beta _j$ (given *β*_*j*_) is specified as *N* (*β*_j_, *V*_*j*_), but a normal prior *N* (0, *W*) is specified for the distribution of *β*_*j*_. Here, the variance *W* can be specified as *W* = 0.21^2^ with a 95% belief that the odds ratio is within 1/1.5 to 1.5. Specifications of *W* incorporating effect-MAF dependence are also possible [16]. Accordingly, the ABF is expressed as a ratio of the probability density $f_{0,\,{\mathrm{ABF}}}\left( {\hat \beta _j} \right)$ under *H*_0_ and the probability density $f_{1,\,{\mathrm{ABF}}}\left( {\hat \beta _j} \right)$ under *H*_1_,

1 $${\mathrm{ABF}}\left( {\hat \beta _j} \right) = \, \frac{{f_{0,\,{\mathrm{ABF}}}\left( {\hat \beta _j} \right)}}{{f_{1,\,{\mathrm{ABF}}}\left( {\hat \beta _j} \right)}} = \frac{{\varphi _{0,\,V_j}\left( {\hat \beta _j} \right)}}{{\int_{ - \infty }^\infty \left\{ {\varphi _{\beta ,\,V_j}\left( {\hat \beta _j} \right) \cdot \varphi _{0,\,W}\left( \beta \right)} \right\}d\beta }}\\ = \, \sqrt {\frac{{V_j + W}}{{V_j}}} exp\left( { - \frac{{z_j^2}}{2}\frac{W}{{(V_j + W)}}} \right),$$

where $\varphi _{\mu ,\,\sigma ^2}$ is the density of the normal distribution with mean *μ* and variance *σ*^2^.

### The SP-EBF

For the *j*th SNP, we assume the following two-component mixture model for the marginal distribution of $\hat \beta _j$,

2 $$f\left( {\hat \beta _j} \right) = \left( {1 - \pi } \right)f_0\left( {\hat \beta _j} \right) + \pi f_1\left( {\hat \beta _j} \right),$$

where *π* is the prior probability of *H*_1_, and *f*_0_ and *f*_1_ are the density distributions of $\hat \beta _j$ under *H*_0_ and *H*_1_, respectively. As with the ABF, we employ asymptotic normality for the sampling distribution of $\hat \beta _j$. Accordingly, we specify *N* (0, *V*_*j*_) for *f*_0_ under *H*_0_. In forming *f*_1_ under *H*_1_, we also specify the sampling distribution of $\hat \beta _j$ as *N* (*β*_*j*_, *V*_*j*_) for a given value of *β*_*j*_, but specify a nonparametric distribution, *g*, for the prior distribution of *β*_*j*_. We then obtain the following BF,

3 $$\frac{{f_0\left( {\hat \beta _j} \right)}}{{f_1(\hat \beta _j)}} = \frac{{\varphi _{0,\,V_j}\left( {\hat \beta _j} \right)}}{{\int_{ - \infty }^\infty \left\{ {\varphi _{\beta ,\,V_j}\left( {\hat \beta _j} \right) \cdot g\left( \beta \right)} \right\}d\beta }}$$

We estimate the priors *π* and *g* based on the data, i.e., the empirical Bayes approach. To this end, we apply the smoothing-and-roughening algorithm [22], a form of the expectation–maximization algorithm [8, 11, 20]. We discretize the effect-size distribution *g* into mass point probabilities ${\boldsymbol{p}} = \left( {p_1,p_2, \ldots ,p_B} \right)$ at points, ${\boldsymbol{t}} = \left( {t_1,t_2, \ldots ,t_B} \right)$ (excluding 0). As such, we approximate *f*_1_ (*y*_*j*_), the denominator of Eq. (3) as $f_1({\hat{\beta}}_{j}) \approx \mathop {\sum}\nolimits_k {\varphi _{t_k,\,V_j}\left( {\hat \beta _j} \right) \cdot p_k}$. This discretized prior distribution excludes the probability mass at the zero point of the null hypothesis [23] (see Section S1 of Supplementary Materials for the details of the algorithms).

Another approach to flexible modeling of the effect-size distribution *g* is to specify parametric finite mixture normal distributions whose components have mean zero, but distinct variances [24, 25]. However, this model could not capture components with non-zero mean (small peaks with relatively large effects) as seen in actual effect-size distributions, e.g., those in schizophrenia and coronary artery disease (see “Applications”). Furthermore, as indicated by these distributions, there is no guarantee that actual effect-size distributions are symmetric. In contrast, our method utilizes a nonparametric distribution for *g* to flexibly capture any forms of the effect-size distribution, including asymmetric multimodal distributions.

We then obtain an estimated BF, i.e., the SP-EBF expressed as

$${\mathrm{SPEBF}}\left( {\hat \beta _j} \right) = \frac{{f_0\left( {\hat \beta _j} \right)}}{{\widehat {f_1}(\hat \beta _j)}} = \frac{{\varphi _{0,\,V_j}\left( {\hat \beta _j} \right)}}{{\mathop {\sum }\nolimits_k \varphi _{t_k,\,V_j}\left( {\hat \beta _j} \right) \cdot \widehat {p_k}}}.$$

R code to implement the estimation of the SP-EBF (including the estimation of the hierarchical mixture model in Eq. (2)) is provided in Section S6 of Supplementary Materials. We ascertained superiority of the SP-EBF over the ABF for various forms of the effect-size distribution by simulation experiments (see Section S4 of Supplementary Materials).

## Applications

We investigated the characteristics of the SP-EBF in comparison with the ABF through their applications to a meta-analysis of seven GWAS studies in bipolar disorder [26], consisting of 7482 cases and 9250 controls (see Section S2 of Supplementary Materials). We utilized summary statistic data and MAF available at the Psychiatric Genomics Consortium website (https://www.med.unc.edu/pgc) on 2135,534 SNPs, after excluding those with no information about allele frequency based on the HapMap CEU sample. In Section S3 of Supplementary Materials, we briefly summarize similar results of our analyses of other two GWAS datasets, in schizophrenia and coronary artery disease.

### Estimation of the effect-size distribution

Figure 1 shows an estimate of the nonparametric effect-size distribution (*g*) for the bipolar disorder dataset. The estimated effect-size distribution was greatly different in dispersion from the ABF normal prior *N* (0, *W*) with *W* = 0.21^2^. This result indicates that the ABF prior missed substantial numbers of small effects, while assuming the presence of substantial numbers of large effects that might not actually be present. The estimation also indicated that the form of the effect-size distribution was not normal (note: this was particularly apparent in the other datasets, specifically in schizophrenia and coronary artery disease (see Fig. S4 in Supplementary Materials); the estimated effect-size distributions had very complex forms with multiple peaks).

### Comparison of the SP-EBF and ABF

**Fig. 2:**
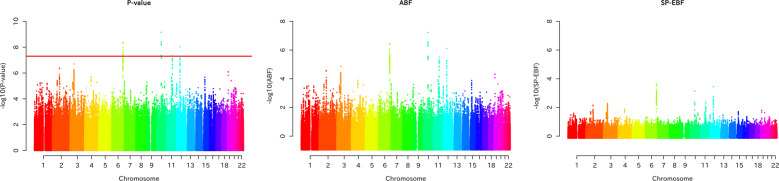
Plots of the P value, ABF, and SBF (–log_10_*P*, –log_10_ ABF, and –log_10_ SP-EBF) for all SNPs, ordered according to the position on the chromosome in the bipolar disorder dataset. Note that the scales in the *P* value plot and those in the BF plots are different, reflecting that *P* value and BF are different measures of association. The red horizontal line in –log_10_ *P* represents the genome-wide significance level.

Figure 2 shows plots of the *P* value, ABF, and SP-EBF across all the SNPs in the bipolar disorder dataset. Note that the scales in the *P* value plot and those in the BF plots are different, reflecting that the *P* value and BF are different measures of association. In the following, for the sake of convenience, we shall use the term “significance” when the *P* value or BFs suggest the alternative hypothesis. At first glance of the SP-EBF plot, the SP-EBF seems to down-weigh the associations consistently for all SNPs, like a zoomed out version of the ABF plot, but actually it is not. The SP-EBF generally down-weighs (or greatly shrinks) the associations for SNPs with very small *P* values, but could up-weigh for those with very small effect-size estimates with large *P* values (with larger supports by the estimated effect-size distribution), according to the shape of the estimated prior distribution shown in Fig. 1. In other words, compared with the SP-EBF, the ABF attributed greater degrees of significance to significant SNPs.

**Fig. 3:**
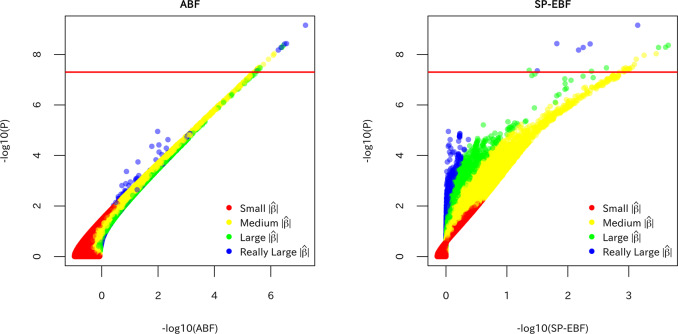
The –log_10_*P* versus the ABF and SP-EBF (–log_10_ ABF and –log_10_ SP-EBF), color-coded by the absolute value of the estimated effect size $\left| {\hat \beta _j} \right|$ in the bipolar disorder dataset; red: small (0–90 percentile), yellow: medium (90–99 percentile), green: large (99–99.9 percentile), blue: very large (99.9–100 percentile). The red horizontal lines in –log_10_ *P* represent the genome-wide significance level. Note that the scales of *x*-axis are different between the ABF and SP-EBF to incorporate the difference in magnitude between them as noted in “Comparison of the SP-EBF and ABF”.

Figure 3 shows scatter plots of the *P* value versus the ABF or SP-EBF for all the SNPs in the bipolar disorder dataset, color-coded by the absolute value of the estimated effect size $\left| {\hat \beta _j} \right|$. In the scatter plots of the *P* value versus the ABF, the points form almost a straight line (this is particularly the case for SNPs with high significance), indicating that the ranking of SNPs using the ABF is almost the same that using the *P* value. In contrast, in the scatter plot for the *P* value versus the SP-EBF, the points are relatively more scattered, indicating a greater difference in SNP ranking between the SP-EBF and the *P* value.

In Fig. 3, as expected by a large difference in the shape of the prior distribution between the ABF and SP-EBF, the ABF and SP-EBF show an opposite tendency in that for a given *P* value, there is a larger –log_10_ ABF (greater significance) for larger $\left| {\hat \beta _j} \right|$ but a larger –log_10_ SP-EBF for smaller $\left| {\hat \beta _j} \right|$. In other words, the SP-EBF ascribed greater significance to SNPs with smaller $\left| {\hat \beta _j} \right|$ (for a given *P* value). We also observed similar results in figures colored based on the variance or MAF (see Figs. S1 and S2 in Supplementary Materials). We observed that for a given *P* value, the SP-EBF attributed greater significance to SNPs with smaller variances or larger MAF. These results are essentially the same as those in Fig. 3, since a small $\left| {\hat \beta _j} \right|$ corresponds to a small standard error or large MAF for a given *P* value (or *z* value).

**Fig. 4:**
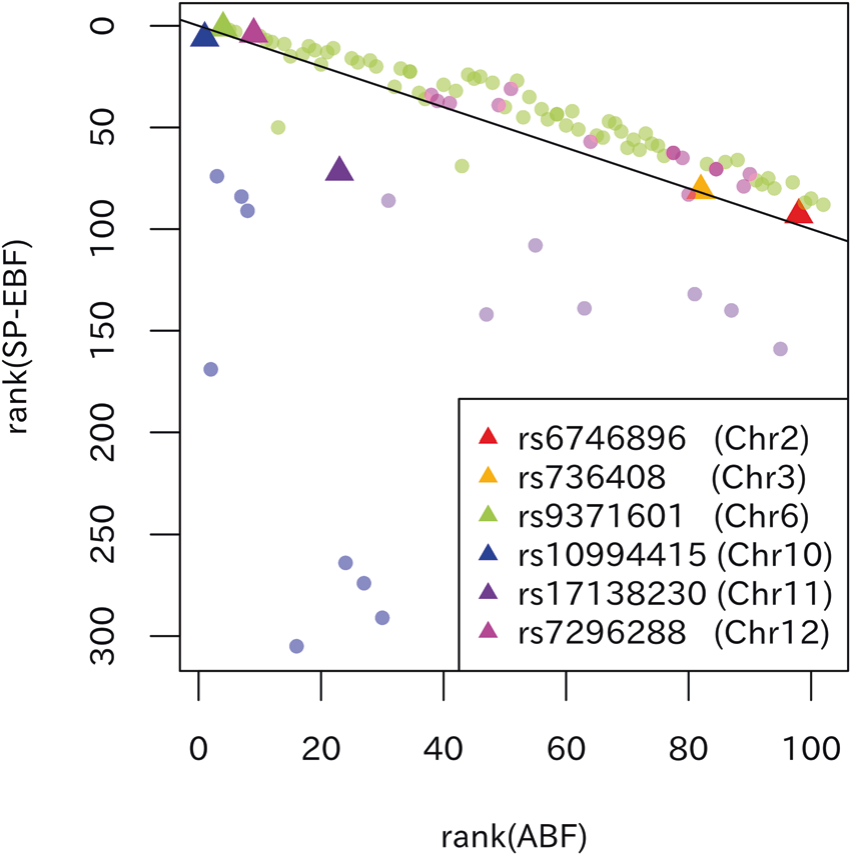
Plot of the ranking in SP-EBF versus that in the ABF for the top 100 SNPs with the smallest *P* values. SNPs in the same linkage disequilibrium region, that had *r*^2^ > 0.2 (according to Haploleg v4.1) or that shared the same GENCODE gene, are plotted using the same color. Representative SNPs (SNPs with the smallest *P* value in each region) are plotted using large dark-colored triangles.

Figure 4 shows a plot of the SP-EBF versus the ABF for the 100 SNPs with the smallest *P* values. These SNPs could be roughly divided into six regions that were in linkage disequilibrium. We observed that SNPs in each region had similar estimated effect sizes. Table 1 presents the SNPs with the smallest *P* values in each of the six regions, and shows their rankings among the top 100 SNPs (without regard to region) based on the ABF, SP-EBF, and *P* value. Again, the rankings based on the ABF and *P* value are almost the same. In comparison, the SP-EBF resulted in a lower ranking of representative SNPs with relatively large $\left| {\hat \beta _j} \right|$ (such as rs10994415 (NC_000010.10:g.62322034T>C) with $\left| {\hat \beta _j} \right|$ = 0.271 and rs17138230 (NC_000011.9:g.79075852A>T) with $\left| {\hat \beta _j} \right|$ = 0.163), and a higher ranking of SNPs with relatively small $\left| {\hat \beta _j} \right|$. Of note, similar results were obtained when dividing SNPs into LD clumps and then comparing the rankings of the associated regions (see Fig. S3 and Table S1). It is interesting to observe that the first-ranked 1 SNP based on the ABF and *P* value, rs10994415, is ranked sixth based on the SP-EBF, while the fourth-ranked SNP based on the ABF and *P* value, rs9371601 (NC_000006.11:g.152790573G>T), is ranked first based on the SP-EBF.

**Table 1:** Representative SNPs from linkage disequilibrium regions.

rsID (Chr)	Absolute value of the estimated effect size $\left| {\hat \beta _j} \right|$	*P* value (rank)	ABF (rank)	SP-EBF (rank)	GENCODE genes
rs10994415 (Chr.10)	0.271	6.97 × 10^−10^ (1)	5.53 × 10^−8^ (1)	7.14 × 10^−4^ (6)	ANK3
rs9371601 (Chr.6)	0.143	4.33 × 10^−9^ (4)	3.76 × 10^−7^ (4)	2.25 × 10^−4^ (1)	SYNE1
rs7296288 (Chr.12)	0.137	9.39 × 10^−9^ (9)	8.13 × 10^−7^ (9)	3.55 × 10^−4^ (4)	3.2 kb 3′ of DHH
rs17138230 (Chr.11)	0.163	4.60 × 10^−8^ (25)	3.28 × 10^−6^ (23)	4.09 × 10^−3^ (72)	ODZ4
rs736408 (Chr.3)	0.134	2.00 × 10^−7^ (82)	1.46 × 10^−5^ (82)	5.16 × 10^−3^ (81)	ITIH3
rs6746896 (Chr.2)	0.127	4.20 × 10^−7^ (98)	2.85 × 10^−5^ (98)	6.85 × 10^−3^ (93)	5.1 kb 5′ of LMAN2L

## Discussion

The applications to real-life GWAS datasets indicated that the ABF prior was excessively dispersed compared to the effect-size distribution estimated by our method (see Figs. 1 and S4). In such situations, it is expected that smaller BFs (more evidence for the alternative hypothesis) to SNPs with smaller effect sizes than those with larger effect sizes, because the estimated effect-size distribution may put more weight on smaller effect sizes. In particular, compared with the SP-EBF, in the ABF a SNP with a large absolute value of the estimated effect size $\left| {\hat \beta _j} \right|$, which is of greater interest in GWASs, may have a larger denominator of the BF (the probability of observing $\hat \beta$ under the alternative hypothesis), leading to a smaller value of the BF (or larger value of –log_10_ BF) (see Fig. 2). That is, the ABF tends to attribute a higher degree of significance to a significant SNP. On the other hand, the ABF may attribute less significance to a SNP with a small $\left| {\hat \beta _j} \right|$ because of the relatively small prior probability assigned to the small absolute value of the estimated effect size.

Another observation in the applications to real-life GWAS datasets was that the SNP rankings were similar between the ABF and *P* value. One reason for this is that the ranking based on *P* value and that based on ${\mathrm{Pr}}\left( {\hat \beta {\mathrm{|H}}_0} \right)$ are generally close (perfectly equal if the estimated variances of $\hat \beta$ are the same across SNPs). Moreover, if the support by a prior effect-size distribution is almost constant (due to its flat form) over an actual range of non-null effect sizes (as indicated by Fig. 1), ${\mathrm{Pr}}\left( {\hat \beta {\mathrm{|H}}_{\mathrm{A}}} \right)$ will be almost constant regardless of the absolute value of the estimated effect size $\left| {\hat \beta _j} \right|$. Therefore, the ABF prior with a large variance *W* essentially functions as a non-informative prior distribution. In other words, it can be said that such a prior distribution may fail to incorporate the information about the alternative hypothesis, although this is the main motivation of using the BF.

On the other hand, the SP-EBF could resolve the aforementioned issues in the ABF by utilizing an actual effect-size distribution estimated under a flexible, semi-parametric hierarchical mixture model. In the applications to real-life GWAS datasets, the estimated effect-size distributions indicated the presence of large numbers of SNPs with small effect sizes. Accordingly, for a SNP with a small absolute value of the estimated effect size $\left| {\hat \beta _j} \right|$, ${\mathrm{Pr}}\left( {\hat \beta {\mathrm{|H}}_{\mathrm{A}}} \right)$ may become larger (due to relatively greater support by the effect-size distribution), leading to a smaller (more significant) BF in the SP-EBF. In contrast, a SNP with a very large absolute value of the estimated effect size would become less significant by using the SP-EBF because of less support by the effect-size distribution. As such, the SP-EBF could successfully incorporate the information about the alternative hypothesis by being based on an actual effect-size distribution.

Based on the arguments above, with the SP-EBF we can expect that SNPs with small effect sizes, where the *P* values are not strongly significant, become more significant and worthy of further investigation in subsequent studies. In the bipolar example, rs6746896 (NC_000002.11:g.97410949A>G) and rs736408 (NC_000003.11:g.52835354C>T) had small effect sizes that did not exceed the genome-wide significance level but that were slightly more significant in the SP-EBF than in the ABF. However, other GWASs [27, 28] reported that bipolar disorder was associated with the gene *LMAN2L*, encoded near rs6746896 (Chr2). Meanwhile, in a pooled population of bipolar and schizophrenia patients, an association was demonstrated [26] with rs736408 (Chr3) in the intron region of *ITIH3*. Of note, for rs10994415, rs9371601, and rs7296288 (NC_000012.11:g.49479968A>C) that exceeded the genome-wide significant level, several studies [29, 30] investigated biological mechanism. The rank improved for rs9371601 for the SP-EBF, although its values were substantially larger than the ABF owing to a less support by the estimated effect-size distribution. The SP-EBF analysis is expected to be particularly useful for detecting novel SNPs with small effect sizes that cannot be detected by standard analysis based on the *P* value, and could therefore address the so called “missing heritability” problem in many complex diseases (see also Nishino et al. [11] and Otani et al. [8]).

Last, as a further extension of our BF analysis, a byproduct of obtaining an estimate, say $\hat \pi$, of the prior probability of null association *π* in Eq. (2), is that it may allow for more accurate Bayesian decision-theoretic testing based on the rule given in “Hypothesis testing and the BF,” utilizing an estimate, $\hat \pi /(1 - \hat \pi ),$ for the prior odds, Pr (*H*_0_)/Pr(*H*_1_). In practice, we may also consider accommodation of stratification factors, permitting possible varying effect-size distributions as well as possible dependence of the probabilities of null association on the stratification factors. See Nishino et al. [11] for stratified analyses based on the derived allele frequency and the status of eQTL. Our method can be easily applied to continuous traits in which a least-square estimate of the slope parameter, rather than the log-odd ratio $\hat \beta$. Compared with empirical Bayes methods under parametric effect-size distributions in the context of human or animal genetic studies [31–33], we can incorporate a nonparametric effect-size distribution into our hierarchical mixture model in Eq. (2) to derive the corresponding SP-EBF such as Eq. (3) (see also Otani et al. [34] for handling continuous traits).

## Supplementary information

Supplemental Materials

get_bf

get_graph

SP-HMM

simulation_cor

simulation_graph

SP-EBF.code

SP-EBF_code_bip

SP-EBF_code_cad

SP-EBF_code_scz

eur
