# Supplementary material for: Semi-parametric empirical Bayes factor for genome-wide association studies
Source: Eur J Hum Genet. 2021 Jan 25;29(5):800–7. doi: 10.1038/s41431-020-00800-x (PMC8110551; doi:10.1038/s41431-020-00800-x)
Supplement: Supplementary file 1 — Supplemental Materials [file 41431_2020_800_MOESM1_ESM.docx]

**Semi-Parametric Empirical Bayes Factor for Genome-wide**

**Association Studies**

**Supplemental Materials**

**Section S1. Estimation Algorithms for the Semi-Parametric Hierarchical Mixture Model**

**S1.1. Expectation-maximization (EM) algorithm**

We estimate the parameters $\pi$ and $g$ in equations (2) and (3) via an EM algorithm^1^. Let $\gamma_{j}$ be the unknown indicator variable for null/non-null status for SNP $j$, such that $\gamma_{j}=1$ if SNP $j$ is non-null and $\gamma_{j}=0$ otherwise. The prior probability of being non-null is $P\left( \gamma_{j}=1 \right)=1-\pi$ and the posterior probability is $P\left( \gamma_{j}=1|\hat{\beta}_{j}=\hat{\beta} \right)={\left( 1-\pi\right)f_{1}\left( \hat{\beta} \right)}/{f\left( \hat{\beta} \right)}$. The posterior probability of being non-null is estimated in the expectation step as

$$\tau_{j}=\frac{\left( 1-\hat{\pi} \right)\hat{f}_{1}\left( \hat{\beta}_{j} \right)}{\hat{\pi}f_{0}\left( \hat{\beta}_{j} \right)+\left( 1-\hat{\pi} \right)\hat{f}_{1}\left( \hat{\beta}_{j} \right)}$$

where $\hat{\pi}$ and $\hat{f}_{1}$ are the empirical estimates of $\pi$ and $f_{1}$. $f_{1}$ is also estimated as

$$\hat{f}_{1}\left( y_{j} \right)=\sum_{k} \varphi_{t_{k},V_{j}}\left( \hat{\beta}_{j} \right)\cdot\hat{p_{k}}.$$

The parameter $\boldsymbol{p}$ (for $g$) and the mixing parameter $\pi$ are estimated in the maximization step as

$$\hat{p_{k}}=\frac{1}{\sum_{j} \tau_{j}}\sum_{j} \tau_{j}\frac{\varphi_{t_{k}, V_{j}}\left( \hat{\beta}_{j} \right)\cdot\hat{p_{k}}}{\sum_{k^{'}} \varphi_{t_{k^{'}},V_{j}}\left( \hat{\beta}_{j} \right)\cdot\hat{p_{k^{'}}}} \left( k=1,\ldots,B \right),$$

and

$$\hat{\pi}=\frac{1}{m}\sum_{j} \left( 1-\tau_{j} \right)$$

respectively.

**S1.2. Estimation guidelines**

Nishino et al.^1^ proposed the following guidelines on the discretization of $g$ in the context of GWAS. The range for the mass points $\boldsymbol{t}$ should be taken sufficiently wide to cover the actual effect-size distributions in complex diseases. For example, they used $\boldsymbol{t}$ = (−0.300, −0.295, …, −0.005, 0.005, …, 0.295, 0.300) with the number grid points as $B=120$. We note that our accumulating experiences of application indicate that the specification of $B$ is generally insensitive to the estimation of $\pi$ and SNP-level statistics (such as posterior means), so that we could specify $B$ within an allowance of computational burden. They also proposed a multi-step implementation of the EM algorithm with update of the initial values for the mass probabilities $\boldsymbol{p}$. With these guidelines, they demonstrated a close agreement of the estimated $\pi$ and $g$ and their true ones in extensive simulation experiments under various scenarios in terms of sample size, $\pi$ and $g$, and the distribution of derived allele frequency (DAF) and correlations across SNPs. We therefore follow their guidelines in this paper. As to computational burden, for a set of summary statistics $\left( \hat{\beta}_{j},V_{j} \right) \left( j=1,\ldots,m \right),$ the estimation algorithm is easily implemented and fast, e.g., < 5 minutes using a personal computer; see Supplementary Materials in Nishino et al^1^).

**Section S2. Analysis of Bipolar Disorder Dataset**

Summary statistic data used in this study are available from the Psychiatric Genomics Consortium website (<https://www.med.unc.edu/pgc>) and the CARDIoGRAMplusC4D Consortium website (<http://www.cardiogramplusc4d.org/>).

In estimating the semi-parametric hierarchical mixture model in (2) we did not include a pre-processing to produce an independent set of SNPs to avoid bias induced by linkage disequilibrium (LD), as done in Nishino et al.^1^. One rationale is given by Otani et al.^2^, where the absence of this process did not affect substantially the estimation with the semi-parametric hierarchical mixture model in an extensive simulation study, where the LD structure was generated according to the results from the 1000 Genome Project. In the application, we assumed a wide range for the effect sizes and specified the mass points as ***t*** = (−0.500, −0.495, …, −0.005, 0.005, …, 0.495, 0.500) in estimating the discretized effect size distributions.

**Supplemental Figures and Tables**


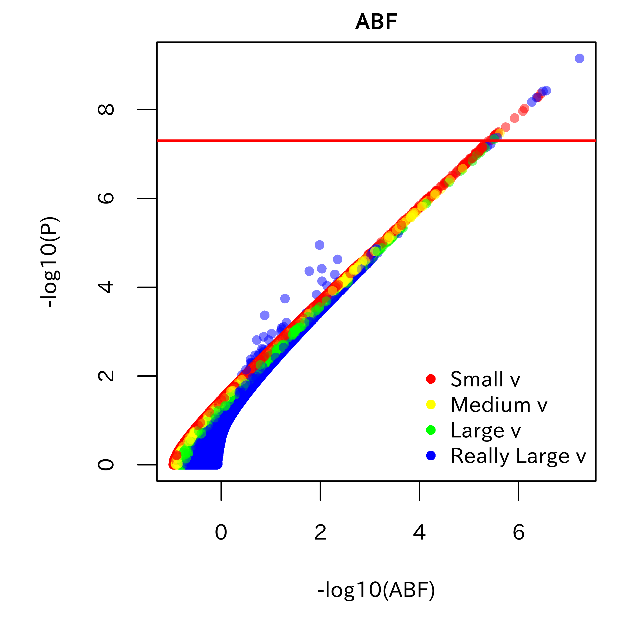

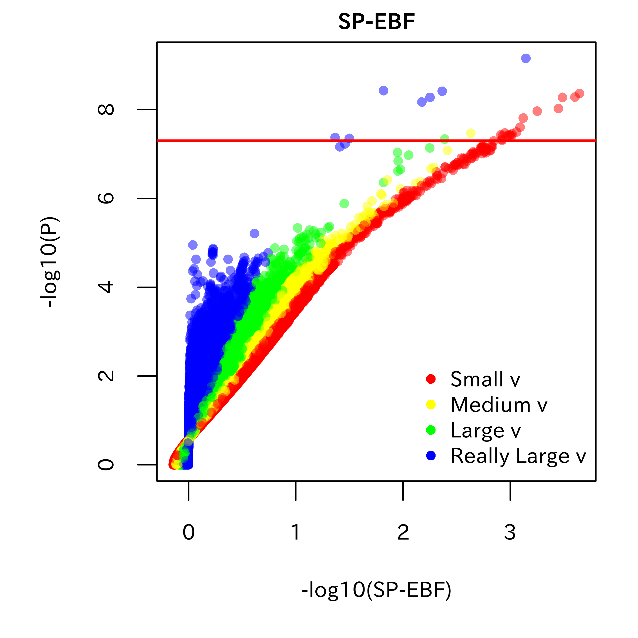


**Figure S1**. The –log_10_ P versus the ABF and SP-EBF (–log_10_ ABF and –log_10_ SP-EBF), color-coded by the variance $V_{j}$ of the effect size estimate in the bipolar disorder dataset; red: small (0-25 percentile), yellow: medium (25-50 percentile), green: large (50-75 percentile), blue: very large (75-100 percentile). The red horizontal lines in –log_10_ P represent the genome-wide significance level. Note that the scales of x-axis are different between the ABF and SP-EBF to incorporate the difference in magnitude between them as noted in Section 3.2.


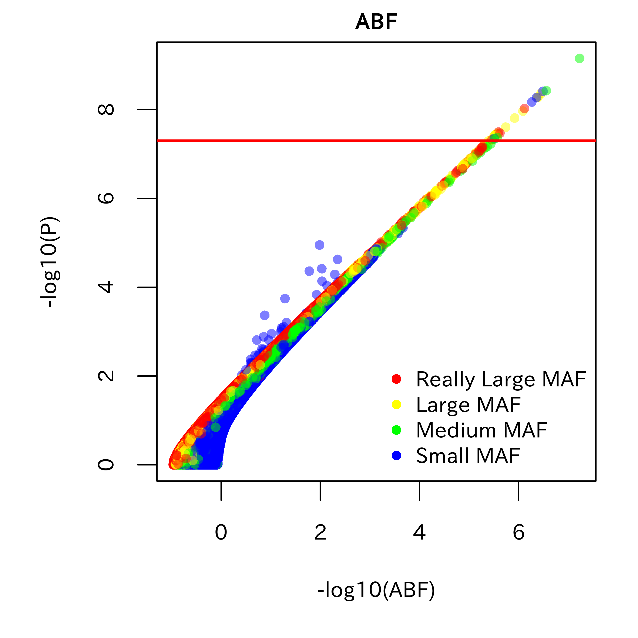

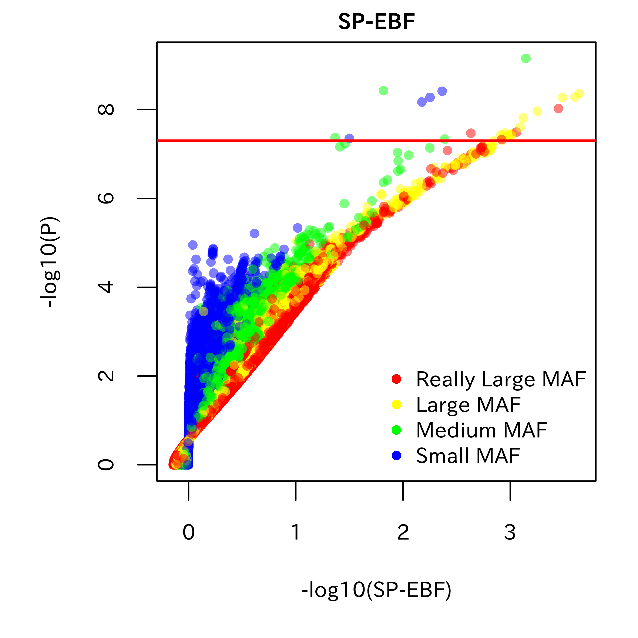


**Figure S2**. The –log_10_ P versus the ABF and SP-EBF (–log_10_ ABF and –log_10_ SP-EBF), color-coded by MAF in the bipolar disorder dataset; red: very large (75-100 percentile), yellow: large (50-75 percentile), green: medium (25-50 percentile), blue: small (0-25 percentile). The red horizontal lines in –log_10_ P represent the genome-wide significance level. Note that the scales of x-axis are different between the ABF and SP-EBF to incorporate the difference in magnitude between them as noted in Section 3.2.


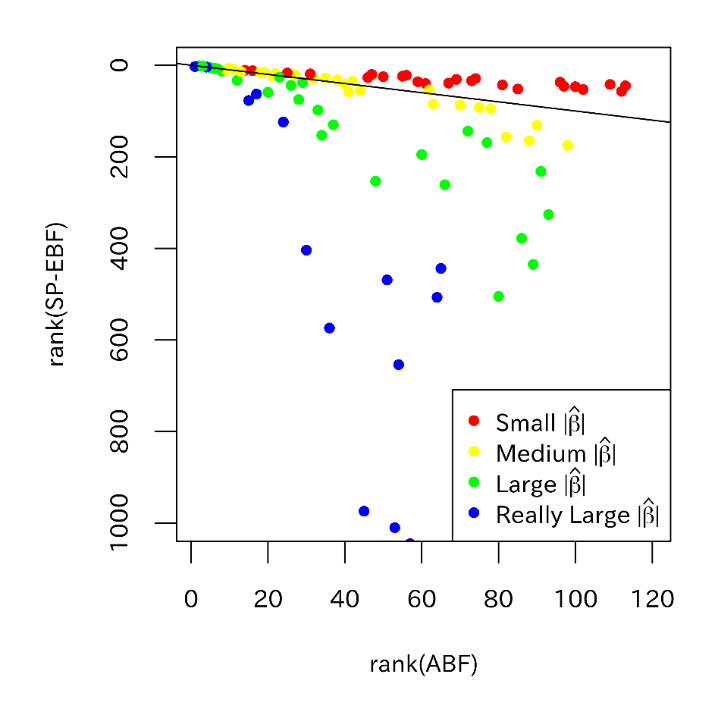


**Figure S3**. Plot of the ranking in SP-EBF versus that in the ABF for the top 100 LD clumps with the smallest P-values. P-value or Bayes factor informed LD clumping (within 500kb window, $r^{2}>0.1$ in 1000 Genomes Phase 1 European population) was done with plink v1.90b6.20.

**Table S1**. Representative SNPs from linkage disequilibrium regions. The rankings were obtained after P-value or Bayes factor informed LD clumping (within 500kb window, $r^{2}>0.1$ in 1000 Genomes Phase 1 European population) with plink v1.90b6.20.

| rsID  （Chr） | Absolute value of the Estimated Effect size $\left\vert\hat{\beta}_{j} \right\vert$ | P-value  （Rank） | ABF  （Rank） | SP-EBF  （Rank） | GENCODE genes |
| --- | --- | --- | --- | --- | --- |
| rs10994415  （Chr.10） | 0.271 | $6.97\times{10}^{-10}$  （1） | $5.53\times{10}^{-8}$  （1） | $7.14\times{10}^{-4}$  （3） | ANK3 |
| rs9371601  （Chr.6） | 0.143 | $4.33\times{10}^{-9}$  （2） | $3.76\times{10}^{-7}$  （2） | $2.25\times{10}^{-4}$  （1） | SYNE1 |
| rs7296288  （Chr.12） | 0.137 | $9.39\times{10}^{-9}$  （3） | $8.13\times{10}^{-7}$  （3） | $3.55\times{10}^{-4}$  （2） | 3.2kb 3’ of DHH |
| rs17138230  （Chr.11） | 0.163 | $4.60\times{10}^{-8}$  （4） | $3.28\times{10}^{-6}$  （4） | $4.09\times{10}^{-3}$  （4） | ODZ4 |
| rs736408  （Chr.3） | 0.134 | $2.00\times{10}^{-7}$  （5） | $1.46\times{10}^{-5}$  （5） | $5.16\times{10}^{-3}$  （5） | ITIH3 |
| rs6746896  （Chr.2） | 0.127 | $4.20\times{10}^{-7}$  （6） | $2.85\times{10}^{-5}$  （6） | $6.85\times{10}^{-3}$  （6） | 5.1kb 5’ of LMAN2L |

**Section S3. Analysis of Schizophrenia and Coronary Artery Disorder Datasets**

We also performed the Bayes factor analyses for two other GWAS datasets, in schizophrenia (34,241 cases and 45,604 controls)^3^ and coronary artery disease (60,801 cases and 123,504 controls)^4^, both of which contained many significant SNPs at the genome-wide significance level. It is known that the degree of polygenicity can vary substantially among complex diseases, especially, extremely high for schizophrenia^5,6^. In fact, in our analysis the estimated proportion of non-null SNPs $\hat{\pi}$ for schizophrenia was 45.1% and greater than those for bipolar disorder and coronary artery disease, 35.3%, and 6.1%, respectively (see Figures 1 and S4). Again, as in the bipolar example, the estimated effect size distributions were much less dispersed than the ABF normal prior and had non-normal forms with some small peaks with large effects (see Figure S4).


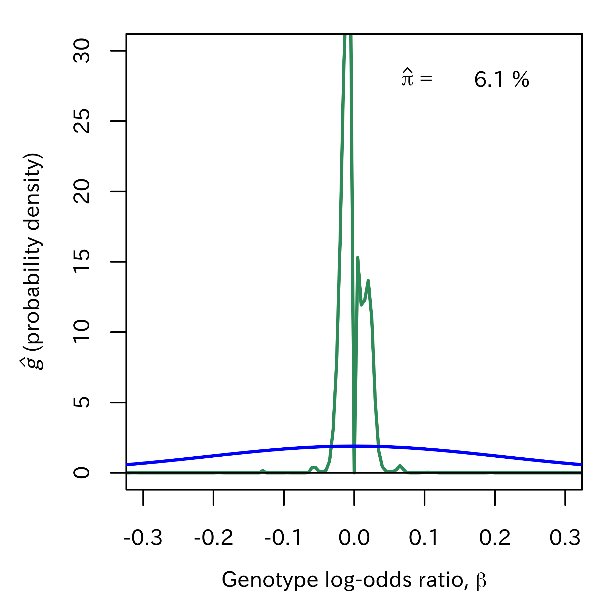

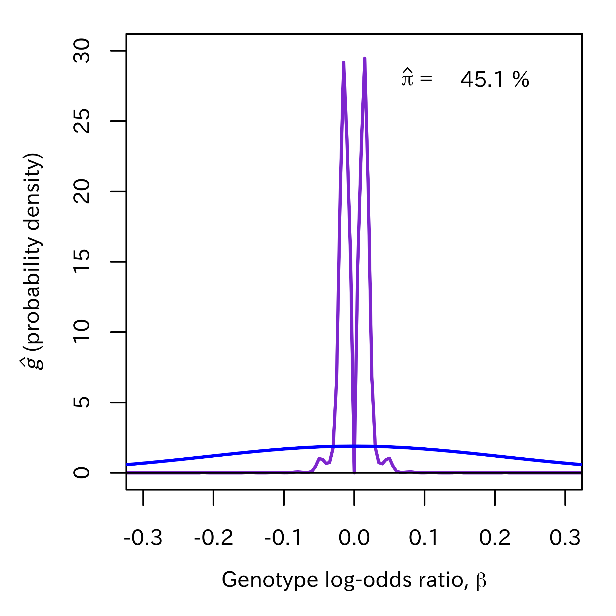


**Figure S4**. The estimated effect size distribution used in the SP-EBF (green line: coronary artery disease; purple line: schizophrenia) and the prior distribution $N\left( 0,W \right)$ with *W* = 0.21^2^ used in the ABF (blue line) in the coronary artery disease and schizophrenia datasets.

In the plots of the P-value versus BF (Figure S5A and S6A), we first observed a very wide range of –log_10_ ABF and –log_10_ SP-EBF, compared with the bipolar example, reflecting the presence of many of highly significant SNPs. The top SNPs showed only a minimal difference in ranking between the SP-EBF and ABF, especially in coronary artery disease, which can be explained by largely distinctive effect sizes across the top SNPs. On the other hand, when focusing on a range of weaker associations around the genome-wide significance level, a comparison between the SP-EBF and ABF demonstrated the same tendency as seen in the bipolar example (Figure S5B and S6B).

1. All SNPs


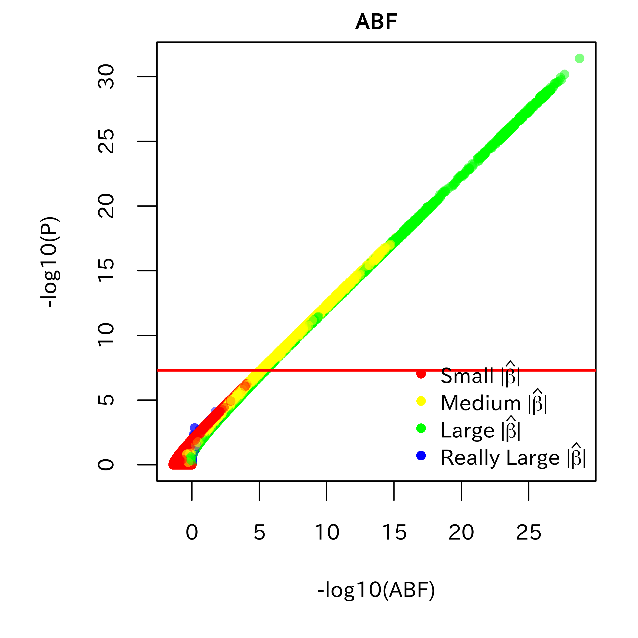

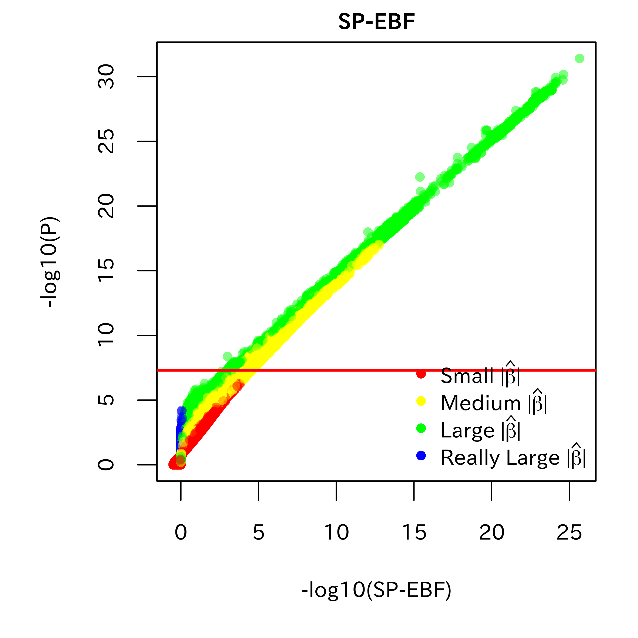


1. SNPs with weaker associations


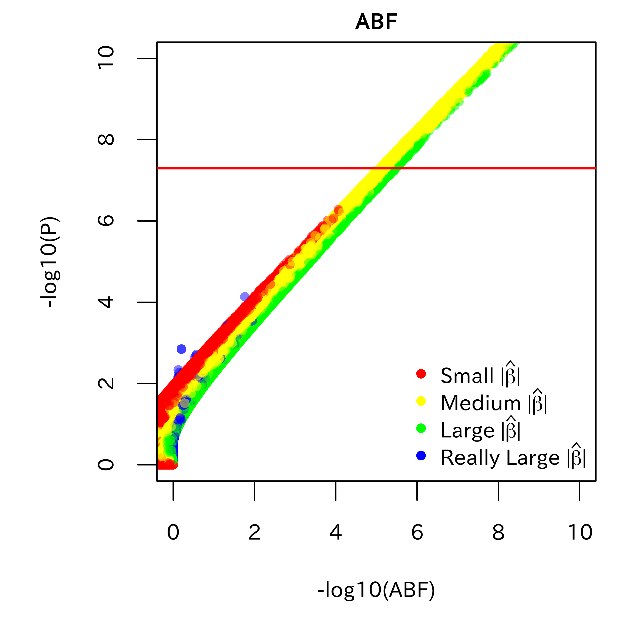

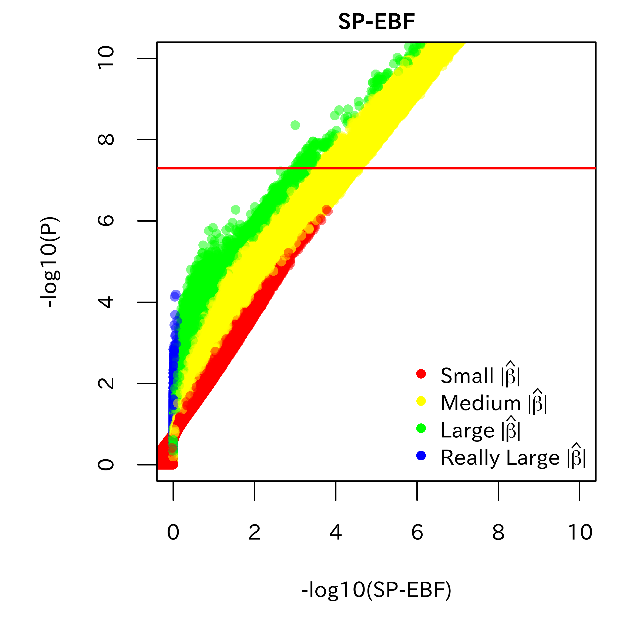


**Figure S5**. The –log_10_ P versus the ABF and SP-EBF (–log_10_ ABF and –log_10_ SP-EBF), color-coded by the absolute value of the estimated effect size $\left| \hat{\beta}_{j} \right|$ in the schizophrenia dataset; red: small (0-90 percentile), yellow: medium (90-99 percentile), green: large (99-99.9 percentile), blue: very large (99.9-100 percentile). The red horizontal lines in –log_10_ P represent the genome-wide significance level. The panels in (A) show plots of all SNPs, with the black frames indicating the SNPs with weaker associations shown in the panels in (B).

1. All SNPs


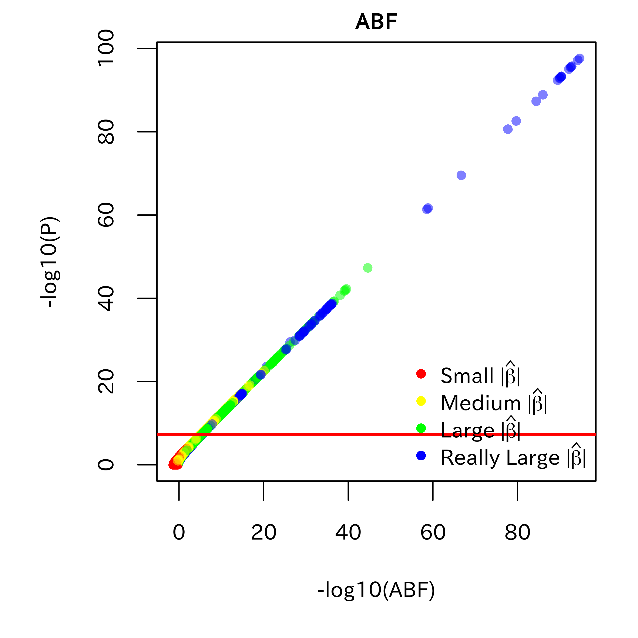

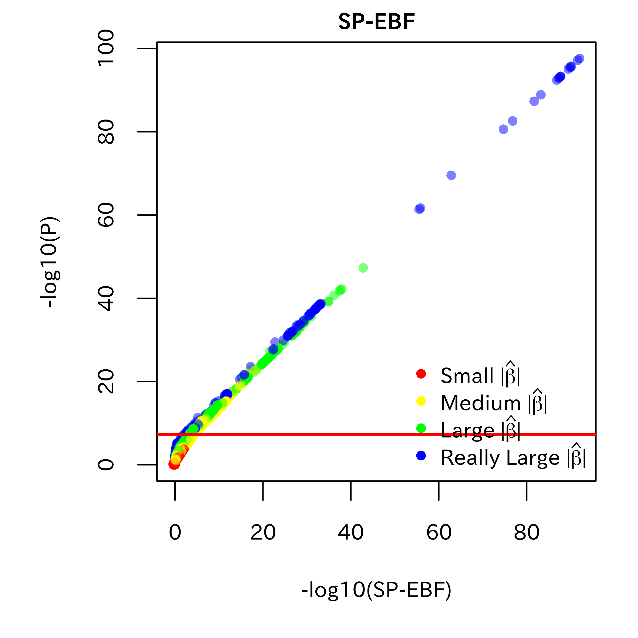


1. SNPs with weaker associations


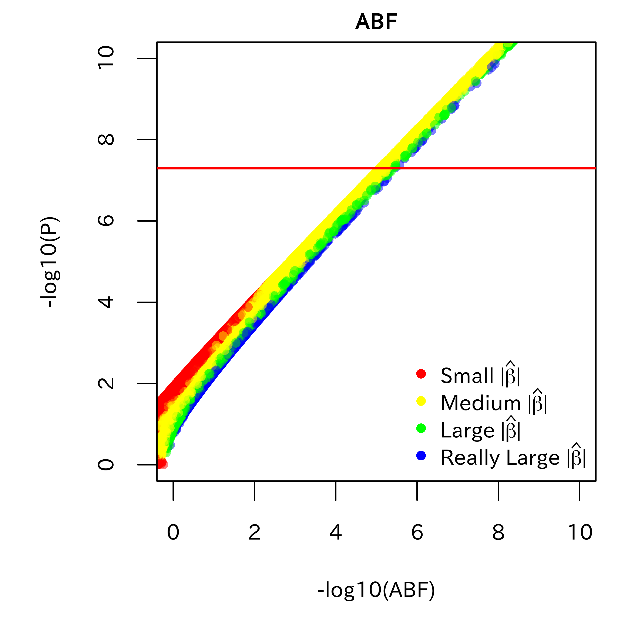

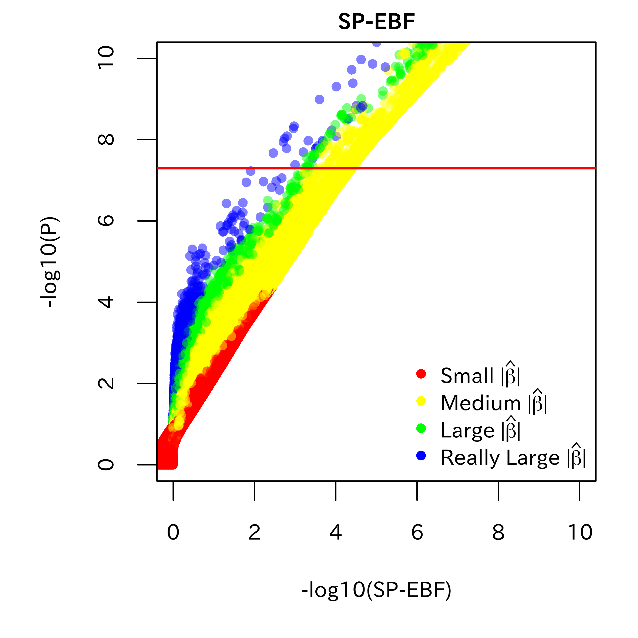


**Figure S6**. The –log_10_ P versus the ABF and SP-EBF (–log_10_ ABF and –log_10_ SP-EBF), color-coded by the absolute value of the estimated effect size $\left| \hat{\beta}_{j} \right|$ in the coronary artery disease dataset; red: small (0-90 percentile), yellow: medium (90-99 percentile), green: large (99-99.9 percentile), blue: very large (99.9-100 percentile). The red horizontal lines in –log_10_ P represent the genome-wide significance level. The panels in (A) show plots of all SNPs, with the black frames indicating the SNPs with weaker associations shown in the panels in (B).

**Section S4. Simulation Experiments**

We conducted a simulation study to evaluate the performance of the SP-EBF and ABF, as well as to confirm the results in Section 3 in the main text. We supposed a GWAS to compare *m* = 1,000,000 SNPs between *n*/2 cases and *n*/2 controls, such that the total number of subjects equals to *n* and the proportion of case $\phi=0.5$. We set *n* as 50,000 or 100,000. We simulated the estimate of log-odds ratio $\hat{\beta}_{j}$based on the hierarchical mixture model in (2) with the two levels of hierarchy. As a first level, we set the prior probability of $H_{1}$ or the proportion of non-null SNPs as $\pi$ = 0.2 or 0.4. As for the effect size distribution $g$ for non-null effect $\beta_{j}$, we assumed a four-component mixture model,

$g \sim\pi_{1}N\left( \theta_{1},W_{1} \right)+\pi_{2}N\left( \theta_{2},W_{2} \right)+\pi_{3}N\left( \theta_{3},W_{3} \right)+\pi_{4}N\left( \theta_{4},W_{4} \right). (S1)$

This model involves various distributional forms as summarized in Table S2, including G1: the normal prior $N\left( 0,W \right)$ by Wakefield (2012)^7^ and G2: a mixture of zero-mean normal distributions, such as those by Zhou et al. (2013)^8^. These distributions are unimodal and symmetric around zero. We also considered more complex forms, G3: multimodal, but symmetric around zero and G4: multimodal and asymmetric around zero, as indicated by the analysis of real GWAS datasets in Section 3.

**Table S2.** Various forms of the effect size distribution in simulations.

| Effect size distribution | $\left( \pi_{1},\pi_{2},\pi_{3},\pi_{4} \right)$ | $\left( \theta_{1},\theta_{2},\theta_{3},\theta_{4} \right)$ | $\left( W_{1},W_{2},W_{3},W_{4} \right)$ |
| --- | --- | --- | --- |
| G1: Wakefield’s prior | $\left( 1, 0, 0, 0 \right)$ | $\left( 0, 0, 0, 0 \right)$ | $\left( {0.21}^{2}, 0, 0, 0 \right)$ |
| G2: Zero-mean normal mixture | $\left( 0.5, 0.3, 0.15, 0.05 \right)$ | $\left( 0, 0, 0, 0 \right)$ | $\left( {0.01}^{2},{0.02}^{2}, {0.05}^{2},{0.1}^{2} \right)$ |
| G3: Multimodal, Symmetric | $\left( 0.5, 0.3, 0.1, 0.1 \right)$ | $\left( 0, 0, -0.05, 0.05 \right)$ | $\left( {0.01}^{2},{0.02}^{2}, {0.01}^{2},{0.01}^{2} \right)$ |
| G4: Multimodal, Asymmetric | $\left( 0.5, 0.3, 0.18, 0.02 \right)$ | $\left( 0, 0, -0.05, 0.05 \right)$ | $\left( {0.01}^{2},{0.02}^{2}, {0.01}^{2},{0.01}^{2} \right)$ |

Given a simulated value for $\beta_{j}$ from the first level, we proceeded to random number generation at a second level of the hierarchical model. Specifically, we simulated $\hat{\beta}_{j}$ from $N\left( \beta_{j},V_{j} \right)$. Here, we set $\beta_{j}=0$ for null SNPs. With respect to $V_{j}$, the variance of $\hat{\beta}_{j}$, we obtained it based on the relation, $V=\left[ 2n\phi(1-\phi)\times maf\times(1-maf) \right]^{-1}$, with MAF, which was simulated by inverse gamma distributions with shape parameter of 2. We considered situations with smaller MAF for larger $|\beta_{j}|$. Specifically, we denote the scale parameter for the set of null SNPs with size $m(1-\pi)$ by $\rho_{null}$. For the set of non-null SNPs with size $m\pi$, we divided them into two subsets with equal size $m\pi/2$ based on the size of $|\beta_{j}|$. We denote the scale parameters for the subset with smaller effects and that with large effects by $\rho_{small}$and $\rho_{large}$, respectively, satisfying that $\rho_{null}<\rho_{small}<\rho_{large}$. We considered two scenarios, $\left( \rho_{null},\rho_{small},\rho_{large} \right)=(50,60,70)$ or$(30,60,90)$. We repeated 20 simulations (20 GWASs data) for each configuration.

For simulated datasets ${(\hat{\beta}_{j},V}_{j}) (j=1,\ldots,m)$, we calculated the ABF and SP-EBF. We also evaluated the “true” BF (TBF) under the true effect size distribution in (S1) above,

$$\mathrm{TBF}\left( \hat{\beta}_{j} \right)= \frac{\varphi_{0,V_{j}}\left( \hat{\beta}_{j} \right)}{\int_{-\infty}^{\infty} \left\{ \varphi_{\beta,V_{j}}\left( \hat{\beta}_{j} \right)\cdot s\left( \beta\right)\text{ } \right\}d\beta}=\frac{N\left( \hat{\beta}_{j};0,V_{j} \right)}{\sum_{k=1}^{4} \pi_{k}N\left( \hat{\beta}_{j};\theta_{k},V_{j}+W_{k} \right)}.$$

We first ascertained the estimation of $\pi$ and $g$ using the EM algorithm (see Figure S7).

1. *n* = 50,00, *p*＝0.2, $\left( \rho_{null},\rho_{small},\rho_{large} \right)$= (30,60,90)

1. *n* = 100,000, *p*＝0.4, $\left( \rho_{null},\rho_{small},\rho_{large} \right)$= (50,60,70)

**Figure S7**. Typical results in estimating *π* and *g* in the simulations. For each configuration, we average the estimated *π* and *g* over 20 simulations. The blue curves pertain to the specified true distribution. The red and green curves pertain to the estimated distributions by the semi-parametric and parametric normal hierarchical mixture models, respectively. See Section S5 in Supplemental Materials for the parametric normal hierarchical mixture model.

The estimated $\pi$ was generally smaller than the specified true value 0.2 or 0.4, especially under scenarios with for G2-G4, reflecting the substantial overlap of the non-null distribution $g$ with the null point because $g$ has the highest peak at zero. Generally, the estimated $g$ showed some fluctuation around the true one, especially, under the smaller sample size *n* = 50,000, but the accuracy improved for the larger sample size *n* = 100,000.

We then compared average correlations among ABF, SP-EBF, and TBF across 20 simulations for top 500 SNPs with greatest absolute effect sizes, summarized in Table S3.

**Table S3.** Average Spearman's rank correlations among ABF, SP-EBF, and TBF across 20 simulations for top 500 SNPs with greatest effect sizes.

| *n* | $\pi$ | ($\rho_{null},\rho_{small},\rho_{large}$) | *g** | ABF vs TBF | SP-EBF vs  TBF | ABF vs  SP-EBF |
| --- | --- | --- | --- | --- | --- | --- |
| 50,000 | 0.2 | (50, 60, 70) | G1 | 1.0000 | 0.9901 | 0.9901 |
|  |  |  | G2 | 0.9700 | 0.9996 | 0.9727 |
|  |  |  | G3 | 0.7335 | 0.9981 | 0.7475 |
|  |  |  | G4 | 0.7271 | 0.9979 | 0.7481 |
|  |  | (30, 60, 90) | G1 | 1.0000 | 0.9900 | 0.9900 |
|  |  |  | G2 | 0.9664 | 0.9982 | 0.9766 |
|  |  |  | G3 | 0.7270 | 0.9948 | 0.7682 |
|  |  |  | G4 | 0.7131 | 0.9928 | 0.7558 |
|  | 0.4 | (50, 60, 70) | G1 | 1.0000 | 0.9853 | 0.9853 |
|  |  |  | G2 | 0.9782 | 0.9998 | 0.9790 |
|  |  |  | G3 | 0.7911 | 0.9983 | 0.8150 |
|  |  |  | G4 | 0.7690 | 0.9978 | 0.7964 |
|  |  | (30, 60, 90) | G1 | 1.0000 | 0.9880 | 0.9880 |
|  |  |  | G2 | 0.9764 | 0.9995 | 0.9800 |
|  |  |  | G3 | 0.7738 | 0.9942 | 0.8307 |
|  |  |  | G4 | 0.7564 | 0.9925 | 0.8130 |
| 100,000 | 0.2 | (50, 60, 70) | G1 | 1.0000 | 0.9907 | 0.9907 |
|  |  |  | G2 | 0.9906 | 0.9999 | 0.9908 |
|  |  |  | G3 | 0.8977 | 0.9990 | 0.9117 |
|  |  |  | G4 | 0.8809 | 0.9990 | 0.8906 |
|  |  | (30, 60, 90) | G1 | 1.0000 | 0.9904 | 0.9904 |
|  |  |  | G2 | 0.9893 | 0.9998 | 0.9906 |
|  |  |  | G3 | 0.8767 | 0.9943 | 0.9174 |
|  |  |  | G4 | 0.8695 | 0.9945 | 0.9011 |
|  | 0.4 | (50, 60, 70) | G1 | 1.0000 | 0.9893 | 0.9893 |
|  |  |  | G2 | 0.9938 | 0.9999 | 0.9939 |
|  |  |  | G3 | 0.9071 | 0.9993 | 0.9193 |
|  |  |  | G4 | 0.8959 | 0.9993 | 0.9054 |
|  |  | (30, 60, 90) | G1 | 1.0000 | 0.9891 | 0.9891 |
|  |  |  | G2 | 0.9933 | 0.9999 | 0.9934 |
|  |  |  | G3 | 0.9040 | 0.9961 | 0.9343 |
|  |  |  | G4 | 0.8907 | 0.9960 | 0.9167 |

*G1: Wakefield’s prior, G2: Zero-mean normal mixture, G3: Multimodal, Symmetric, G4: Multimodal, Asymmetric.

The ABF was completely correlated with the TBF (correlation coefficient = 1) under the G1 (Wakefield’s) effect size distribution, as expected, but the correlation reduced for more complicated, G2-G4 forms of the effect size distribution. In contrast, the correlation between SP-EBF and TBF was very stable with very high correlations ($\geq$ 0.99) for almost all the forms of the effect size distribution. Note that the correlations for top 500 SNPs were very stable irrespective of *n*, even when the estimation of $\pi$ and $g$ was relatively unstable when *n* was small. The correlation between the SP-EBF and ABF was very high under the G1 effect size distribution, but reduced under the others distributions that deviate from G1.

Figure S8 shows scatter plots from one simulation set ${(\hat{\beta}_{j},V}_{j}) \left( j=1,\ldots,m \right)$ under the G4 effect size distribution (multimodal and asymmetric) when *n* = 100,000, $\pi$ = 0.2, and ($\rho_{null},\rho_{small},\rho_{large}$) =(50, 60, 70).


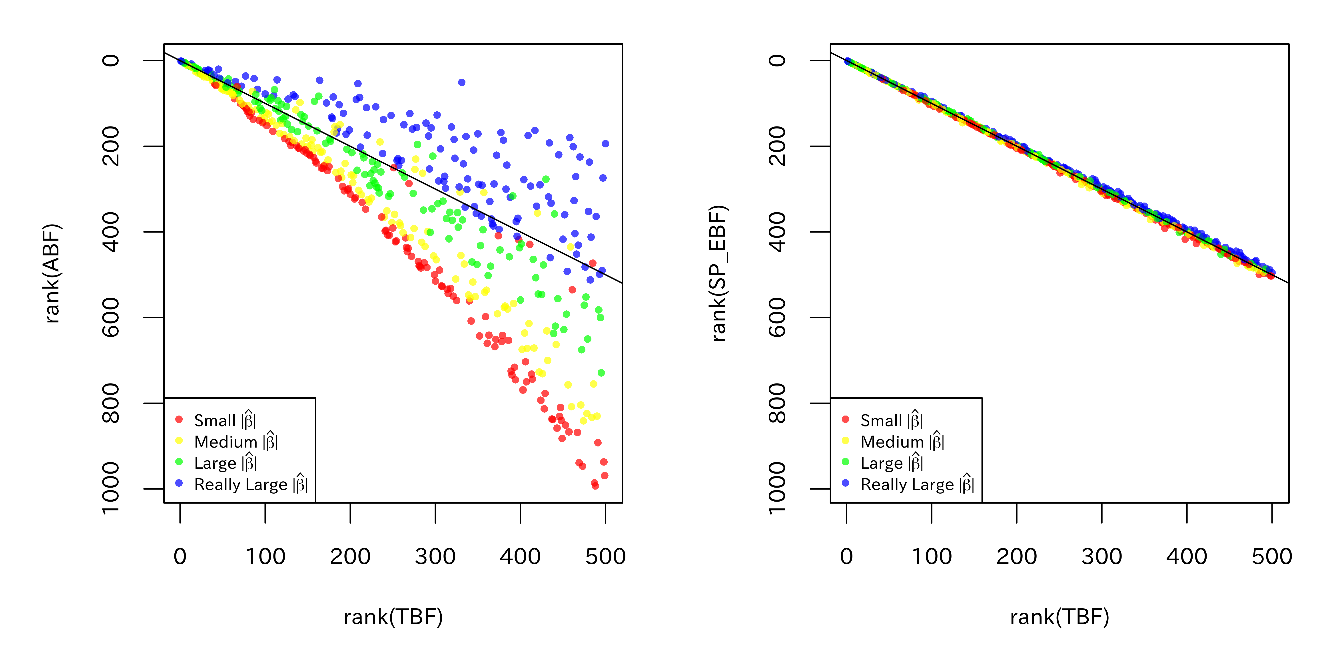


**Figure S8.** Scatter plot of the ranking in ABF versus that in the TBF (left) and the ranking in the SP-EBF versus that in the TBF (right) for top 500 SNPs with smallest TBF under the scenario with *n* = 100,000, $\pi$ = 0.2, and ($\rho_{null},\rho_{small},\rho_{large}$) =(50, 60, 70).

The ABF tended to give higher ranks for SNPs with larger absolute values of the estimated effect size $|\hat{\beta}_{j}|$, as observed in Section 3 in the main text, while the SP-EBF provided very accurate SNP ranking irrespective of the effect size estimates for top 500 SNPs. As for direct comparison between the ABF and SP-EBF, Figure S9 shows scatter plots of the ABF or SP-EBF versus the P-value for all the SNPs, color-coded by $\left| \hat{\beta}_{j} \right|$ or variance estimate $V_{j}.$


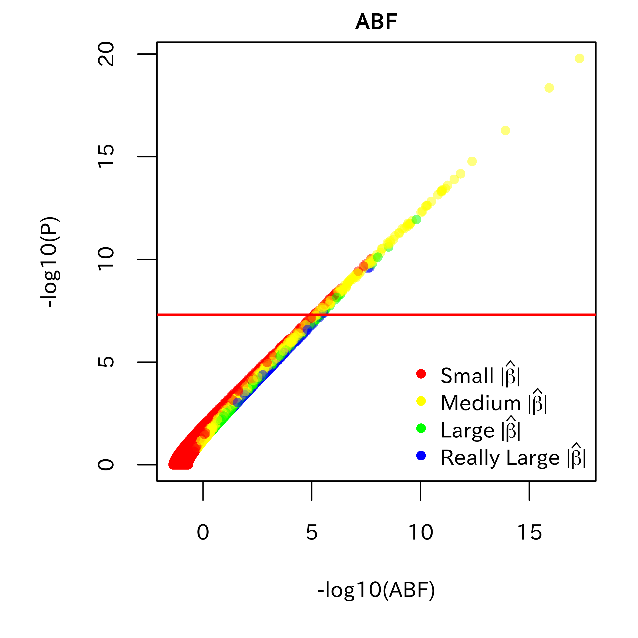

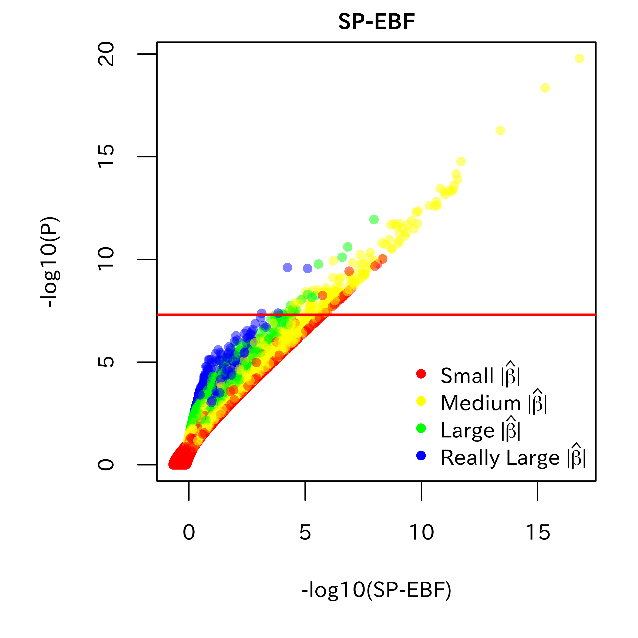

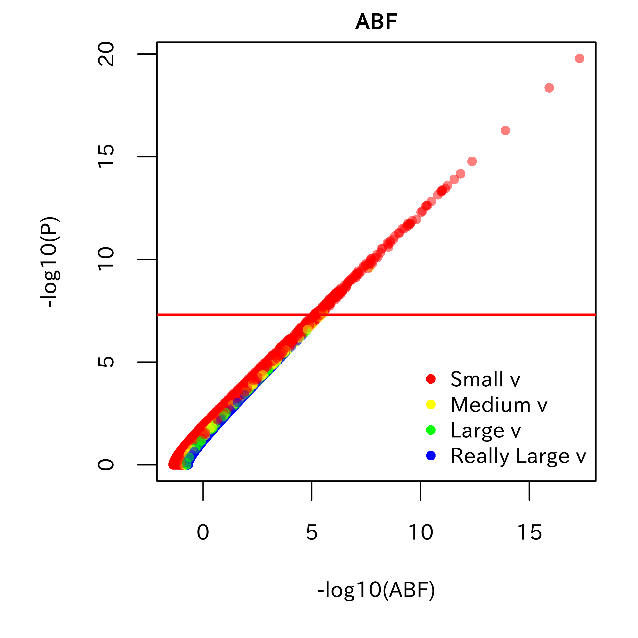

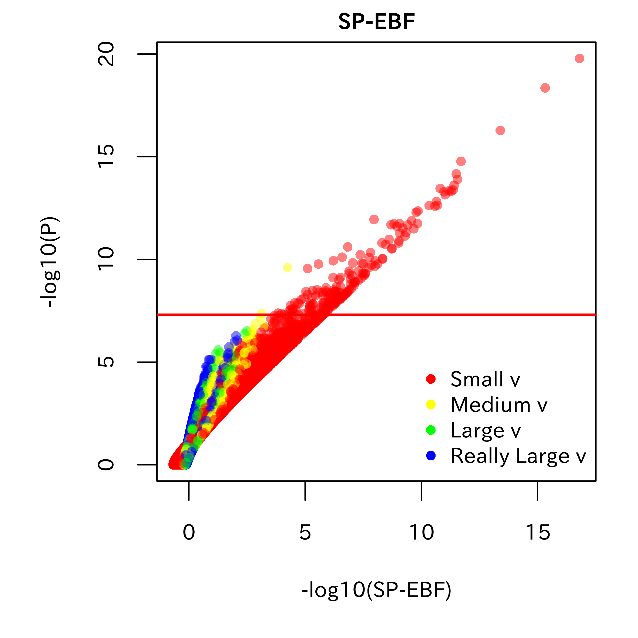


**Figure S9**. The –log_10_ P versus the ABF and SP-EBF (–log_10_ ABF and –log_10_ SP-EBF), color-coded by the absolute value of the estimated effect size $\left| \hat{\beta}_{j} \right|$ (upper panels) or variance estimate $V_{j}$ (lower panels) in simulation under the G4 effect size distribution (*n* = 100,000, $\pi$ = 0.2, ($\rho_{null},\rho_{small},\rho_{large}$) =(50, 60, 70)). Four groups based on the estimated effect size are red: small (0-90 percentile), yellow: medium (90-99 percentile), green: large (99-99.9 percentile), blue: very large (99.9-100 percentile). Four groups based on the variance estimate are red: small (0-25 percentile), yellow: medium (25-50 percentile), green: large (50-75 percentile), blue: very large (75-100 percentile). The red horizontal lines in –log_10_ P represent the genome-wide significance level.

We ascertained the same tendencies observed in Figure 3 in the main text. The ABF and SP-EBF show an opposite tendency in that for a given P-value, there is a larger –log_10_ ABF (greater significance) for larger $\left| \hat{\beta}_{j} \right|$ but a larger –log_10_ SP-EBF for smaller $\left| \hat{\beta}_{j} \right|$. And, for a given P-value, the SP-EBF attributed greater significance to SNPs with smaller variance.

Following a suggestion from a reviewer, we also evaluated a parametric normal empirical Bayes factor (PN-EBF) that estimates the variance $W$ of the normal prior $N\left( 0,W \right)$ specified in the ABF, rather than estimating the non-parametric prior as in the SP-EBF. See Section S5 of Supplemental Materials for the estimation algorithm in the framework of our hierarchical mixture model and simulation results for comparison. In summary, as expected, the PN-EBF performed better than the ABF, especially under the scenarios G2-G4 (whose effect size distributions deviate from $N\left( 0,W \right)$), but the improvement by estimating $W$ could be limited under scenarios G3 and G4 with non-normal effect size distributions.

**Section S5: Empirical Bayes Factor with Parametric Normal Hierarchical Mixture Model**

**S5.1 The model and estimation algorithm**

We also considered another empirical Bayes factor based on a hierarchical mixture model with the parametric normal distribution *N*(0, *W*) (i.e., Wakefield’s prior) for *g*, called PN-EBF, rather than the SP-EBF with a non-parametric distribution for *g*.

We can tailor the EM-algorithm in the following way to estimate the variance *W* based on the data. Again, $\hat{\beta}_{j}\sim N\left( 0,V_{j} \right)$ under ${H_{0}: \beta}_{j}=0$ and $\hat{\beta}_{j}\sim N\left( \beta_{j},V_{j} \right)$ under $H_{1}$: $\beta_{j}\neq0$, and $\beta_{j}\sim N\left( 0,W \right)$. As the marginal distribution, we have $\hat{\beta}_{j}\sim N\left( 0, V_{j}+W \right)$. We define an indicator variable $\theta_{j}$, such that $\theta_{j}=1$ if $H_{1}$ is true for the *j*-th SNP and $\theta_{j}=0$otherwise. And, let$\pi$ be the prior probability that $H_{1}$ is true, $\pi=\Pr\left( \theta_{j}=1 \right)$. The likelihood for the parameter $\varphi=(\pi,W)$ given the observed data, $\boldsymbol{b}=(\hat{\beta}_{j};j=1,\ldots,m)$, using $\boldsymbol{\theta}=\left( \theta_{j};j=1,\ldots,m \right)$ is given by

$$L\left( \varphi: \boldsymbol{b} \right)=log\left( p\left( \boldsymbol{b} | \varphi\right) \right)=log\left( p\left( \boldsymbol{b, \theta} | \varphi\right) \right)-log\left( p\left( \boldsymbol{\theta} | \boldsymbol{b;}\varphi\right) \right),$$

where

$$p(\boldsymbol{\theta|b;}\varphi)=\prod_{j} \left\{ p(\hat{\beta}_{j}|\theta_{j}, \varphi)p(\theta_{j}|\varphi) \right\}/\left\{ p(\hat{\beta}_{j}|\varphi) \right\}$$

$$p\left( \boldsymbol{b, \theta} | \varphi\right)=\prod_{j} \left. p\left( \hat{\beta}_{j}, \theta_{j} | \varphi\right) \right.=\prod_{j} \left\{ \left( 1-\pi\right)f_{0}\left( \hat{\beta}_{j} \right) \right\}^{1-\theta_{j}}\left\{ \pi f_{1}\left( \hat{\beta}_{j} \right) \right\}^{\theta_{j}}$$

For $\varphi^{(t)}$ as the *t*-th updated parameter value for$\varphi$, the EM algorithm maximizes the following function,

$$Q(\varphi| \varphi^{\left( t \right)}) = E[log(p(\boldsymbol{B, \theta}|\varphi)) | \boldsymbol{B} = \boldsymbol{b}; \varphi^{\left( t \right)}]$$

$$=\sum_{j} \sum_{\theta_{j}} p(\theta_{j}|\hat{\beta}_{j}, \varphi^{\left( t \right)})[(1-\theta_{j})\{log(1-\pi)+logf_{0}\left( \hat{\beta}_{j} \right)\}+\theta_{j}\{log(\pi)+logf_{1}\left( \hat{\beta}_{j} \right)\}]$$

With ${\lambda_{j}}^{(t)}(\theta_{j}) = \left\{ p(\hat{\beta}_{j}| \theta_{j},\varphi^{(t)})p(\theta_{j}|\varphi^{(t)}) \right\}/\left\{ p(\hat{\beta}_{j}|\varphi^{(t)}) \right\}$, the formulas for updating are obtained by differentiating $\pi$ or *W* as:

$$\pi= \frac{1}{m}\sum_{j} {\lambda_{j}}^{(t)}(1)$$

$$\sum_{j} {\lambda_{j}}^{(t)}(1)\frac{1}{V_{j}-W} = \sum_{j} {\lambda_{j}}^{(t)}(1)\frac{{y_{j}}^{2}}{{(V_{j}-W)}^{2}}$$

**S5.2 Simulation results**

We evaluated the performance of the PN-EBF in the simulation study in Section S4. Figure S7 shows the effect size distribution with the estimated *W* (green curves), indicating that the estimated distribution captures the true one very well under the G1 effect size distribution (i.e., Wakefield’s prior), but does not so under the other distributions that deviate from the G1 distribution. The average correlations between PN-EBF and TBF across 20 simulations for top 500 SNPs with greatest absolute effect sizes, summarized in Table S4, indicated that the correlation is very high under the G1 effect size distribution, but can drop down under the other distributions that deviate from the G1 distribution. Overall, the PN-EBF is sensitive to the form of the underlying effect size distribution. On the other hand, the SP-EBF generally performed better, even when the underlying effect size distribution deviated from the G1 distribution.

**Table S4.** Average Spearman's rank correlations between ABF, SP-EBF, or PN-EBF versus TBF across 20 simulations for top 500 SNPs with greatest effect sizes.

| *n* | $\pi$ | ($\rho_{null},\rho_{small},\rho_{large}$) | *g** | ABF vs TBF | SP-EBF vs  TBF | PN-EBF vs  TBF |
| --- | --- | --- | --- | --- | --- | --- |
| 50,000 | 0.2 | (50, 60, 70) | G1 | 1.0000 | 0.9901 | 1.0000 |
|  |  |  | G2 | 0.9700 | 0.9996 | 0.9954 |
|  |  |  | G3 | 0.7335 | 0.9981 | 0.9941 |
|  |  |  | G4 | 0.7271 | 0.9979 | 0.9522 |
|  |  | (30, 60, 90) | G1 | 1.0000 | 0.9900 | 1.0000 |
|  |  |  | G2 | 0.9664 | 0.9982 | 0.9980 |
|  |  |  | G3 | 0.7270 | 0.9948 | 0.9928 |
|  |  |  | G4 | 0.7131 | 0.9928 | 0.9524 |
|  | 0.4 | (50, 60, 70) | G1 | 1.0000 | 0.9853 | 1.0000 |
|  |  |  | G2 | 0.9782 | 0.9998 | 0.9925 |
|  |  |  | G3 | 0.7911 | 0.9983 | 0.9908 |
|  |  |  | G4 | 0.7690 | 0.9978 | 0.9569 |
|  |  | (30, 60, 90) | G1 | 1.0000 | 0.9880 | 1.0000 |
|  |  |  | G2 | 0.9764 | 0.9995 | 0.9962 |
|  |  |  | G3 | 0.7738 | 0.9942 | 0.9873 |
|  |  |  | G4 | 0.7564 | 0.9925 | 0.9527 |
| 100,000 | 0.2 | (50, 60, 70) | G1 | 1.0000 | 0.9907 | 1.0000 |
|  |  |  | G2 | 0.9906 | 0.9999 | 0.9966 |
|  |  |  | G3 | 0.8977 | 0.9990 | 0.9932 |
|  |  |  | G4 | 0.8809 | 0.9990 | 0.9718 |
|  |  | (30, 60, 90) | G1 | 1.0000 | 0.9904 | 1.0000 |
|  |  |  | G2 | 0.9893 | 0.9998 | 0.9983 |
|  |  |  | G3 | 0.8767 | 0.9943 | 0.9894 |
|  |  |  | G4 | 0.8695 | 0.9945 | 0.9668 |
|  | 0.4 | (50, 60, 70) | G1 | 1.0000 | 0.9893 | 1.0000 |
|  |  |  | G2 | 0.9938 | 0.9999 | 0.9952 |
|  |  |  | G3 | 0.9071 | 0.9993 | 0.9904 |
|  |  |  | G4 | 0.8959 | 0.9993 | 0.9725 |
|  |  | (30, 60, 90) | G1 | 1.0000 | 0.9891 | 1.0000 |
|  |  |  | G2 | 0.9933 | 0.9999 | 0.9969 |
|  |  |  | G3 | 0.9040 | 0.9961 | 0.9871 |
|  |  |  | G4 | 0.8907 | 0.9960 | 0.9684 |

*G1: Wakefield’s prior, G2: Zero-mean normal mixture, G3: Multimodal, Symmetric, G4: Multimodal, Asymmetric.

**Section S6: R codes to Implement the SP-EBF Method**

R codes to implement the SP-EBF method are included in the “SP-EBF.zip” file as follows:

SP-EBF.zip/

├ Bipolar/ …. For analysis of the bipolar disorder GWAS (Sklar et al., 2011)

│ ├ data/

│ ├ graph/

│ └ result/

├ functions/

│ ├ get_bf.R …. Calculate Bayes factor

│ ├ get_graph.R …. Plot graphs utilized in the paper

│ └ SP-HMM_0.5.R …. Expectation–maximization (EM) algorithm

└ SP-EBF_code.R …. For reproduction

**Instructions for replication**

1. Download summary statistic data file “pgc.bip.2012-04.zip” from the Psychiatric Genomics Consortium website (<https://www.med.unc.edu/pgc>).
   1. Open **Download Results** page.
   2. Fill out “Downloads Filter” form. Select “Bipolar Disorder (BIP)” for **PGC Downloads** and for **Secondary Choice**. Then, click **Go to Downloads**.
   3. Fill out “Bipolar Disorder Data Download Agreement” form. For **Dataset**, please select “BIP”. And then, click **Submit**.
   4. Download the file from “PGC Bipolar Disorder Downloads.”
2. Unzip the downloaded file and copy “pgc.bip.full.2012-04.txt” to the “Bipolar/data” directory.
3. Run “SP-EBF_code.R” in R. The figures in the paper will be created in the “Bipolar/graph” directory.

**References**

1. Nishino J, Kochi Y, Shigemizu D, Kato M, Ikari K, Ochi H et al. Empirical Bayes estimation of semi-parametric hierarchical mixture models for unbiased characterization of polygenic disease architectures. *Front Genet* 2018, 9, 115.
2. Otani T, Noma H, Nishino J, Matsui S. Re-assessment of multiple testing strategies for more efficient genome-wide association studies. *Eur J Hum Genet* 2018, 26, 1038–1048.
3. Ripke S, Neale BM, Corvin A, Walters JTR, Farh K, Holmans PA. Biological insights from 108 schizophrenia-associated genetic loci. *Nature* 2014, 511, 421-427.
4. Nikpay M, Goel A, Won H, Hall LM, Willenborg C, Kanoni S et al. A comprehensive 1000 Genomes–based genome-wide association meta-analysis of coronary artery disease. *Nature Genetics* 2015, 47(10), 1121-1130.
5. Stahl E, Wegmann D, Trynka G, Gutierrez-Achury J, Do R, Voight BF et al. Bayesian inference analyses of the polygenic architecture of rheumatoid arthritis. *Nature Genetics* 2012, 44(5), 483-489.
6. Ripke S, O`Dushlaine C, Chambert K, Moran JL, Kähler AK, Akterin S et al. Genome-wide association analysis identifies 13 new risk loci for schizophrenia. *Natare Genetics* 2013, 45(10), 1150–1159.
7. Wakefield J. Bayes Factors for Genome-Wide Association Studies: Comparison with P-values. *Genetic Epidemiology* 2009, 33(1), 79-86.
8. Zhou X, Carbonetto P, Stephens M. Polygenic modeling with Bayesian sparse linear mixed models. *PLoS Genetetics* 2013, 9(2), e1003264.
